# Sporicidal Action of Pulsed Radiation of Hot Plasma

**DOI:** 10.17691/stm2022.14.4.02

**Published:** 2022-07-29

**Authors:** I.M. Piskarev, I.P. Ivanova

**Affiliations:** Leading Researcher, Skobeltsyn Institute of Nuclear Physics; Lomonosov Moscow State University, 1–2 Leninskiye Gory, Moscow, 119234, Russia;; Professor, Molecular Biology and Immunology Department, Institute of Biology and Biomedicine; National Research Lobachevsky State University of Nizhni Novgorod, 23 Prospekt Gagarina, Nizhny Novgorod, 603950, Russia

**Keywords:** hot plasma, UV radiation, L-tyrosine, nitration, biocidal effect, sporicidal effect

## Abstract

Nitration is one of the main reaction mechanisms of long-living reactive species (oxygen and nitrogen products) formed under the action of pulsed radiation of hot plasma. The long lifetime of reactive species formed in a discharge zone (up to several days) enables them to penetrate deep into the objects covered with a protective coat. Spores are such objects.

**The aim of the investigation** was to study sporicidal activity of hot plasma pulsed radiation of spark electric discharge by the analysis findings of active products formed in an aqueous L-tyrosine solution under the effect of the discharge.

**Materials and Methods:**

In the study, we used a Pilimin IR-10 spark discharge generator as a source of pulsed radiation of hot plasma; a corona discharge generator — as a source of cold plasma; a DKB-9 low-pressure mercury lamp — as a source of continuous radiation of UV band, wavelength of 253.7 nm. The samples were processed in Petri dishes, 40 mm in diameter, their volume being 4 and 10 cm^3^. The study used an L-tyrosine solution in distilled water (the concentration: 160 mg/L), a suspension of bacteria and spores of micromycetes (its concentration being ~10^6^ cells per 1 ml). Tyrosine conversion products were identified spectrophotometrically before and after treatment. The biocidal and sporicidal effects were assessed by counting CFU (colony-forming units) after seeding incubation at 27–37°C.

**Results:**

The oxidation of tyrosine by HO_2_^•^ radicals was found to be impossible. Under 2 the action of nitrogen compounds, nitration proceeds with 3-nitrotyrosine formation. The nitration reaction is slow, taking about 100 h. A possible nitration mechanism is through the formation of the nitronium ion NO_2_^+^ in an acidic medium.

The biocidal effect of hot plasma radiation turned out to be weaker than that of UV radiation of a DKB-9 lamp. This is due to the difference in their emission spectrum. The sporicidal effect of hot plasma radiation was more pronounced: a 10-fold decrease in the number of CFU was observed at radiation doses of 200–280 J. Under the action of UV radiation, at the same doses, the decrease in the number of CFU was from 3 to ~30%. The sporicidal effect of hot plasma radiation is due to the decay of a long-living …ONOOH/ONOO^–^… complex with the formation of a nitric oxide and a nitronium ion in an acidic medium.

**Conclusion:**

The study showed the viability of spores under the action of pulsed radiation of hot plasma to decrease. While the light radiation of a UV lamp, under studied conditions, slightly penetrates the protective coating of a spore. The sporicidal effect of hot plasma radiation is due to the decay of a long-living …ONOOH/ONOO^–^… complex with the formation of a nitric oxide and a nitronium ion in an acidic medium. Nitration plays a decisive role in the sporicidal action of the hot plasma radiation of a spark discharge. The principle of the sporicidal effect of gas-discharge plasma radiation can be used to develop disinfecting devices.

## Introduction

Hot plasma of pulsed electric discharge can have an effect on biological and non-biological objects under study at a distance causing no thermal injuries. Plasma radiates as a hot black body with temperature of ~10^4^ К in ultraviolet, visible and red bands, the radiation spectrum maximum being 220 nm. In a discharge zone, reactive species of oxygen and nitrogen form. Reactive oxygen species die in the place they are formed due to high reactive capacity. Nitrogen radicals have longer lifetime (about several seconds) and are able to diffuse to an object. Moreover, nitric oxide exhibits high penetration through biological membranes [1]. When liquid is treated, radicals can be absorbed through the gas–liquid interface. The study [2] showed the role of particles diffusing from a discharge zone to be minor. Plasma radiation was found to play a major role: it both has an effect on the surface and penetrates the liquid at a considerable depth. Therefore, this study will concern hot plasma radiation.

The active forms formed in water under radiation are HO_2_^•^ radicals, hydrogen dioxide H_2_O_2_, nitric acid HNO_2_ and …ONOOH/ONOO^–^… complex formed in water during radiation pulse and segregating within 15 min into peroxynitrites and peroxynitric acid HOONO [3]. Water pH decreases after the treatment due to nitric acid formation. Since for a pair ONOOH/ONOO^–^ an acid dissociation constant pK_a_=6.8, peroxynitric acid plays a key role when a complex disintegrates in an acidic medium.

The main oxidizer formed directly under radiation is a radical HO_2_^•^[3]. Additionally, when peroxynitric acid breaks up, hydroxyl radicals can form. Nitrogen compounds can serve as deoxidants. In case of pulsed radiation of hot plasma has an effect on inorganic substances, the yield of oxidation and deoxidization was found to be nearly the same; an absolute yield value (~5 molecules per 100 eV of consumed energy) is close to the yield in a radiation-chemical process [4]. Therefore, pulsed radiation of hot plasma can be an effective treatment technique for liquids or surfaces in various technological processes.

An effect of cold plasma and a remote effect of hot plasma on aqueous solutions are very much different. Cold plasma is directly in contact with an object under treatment, and active particles penetrate the solution through the gas–liquid interface [5]. The main active particle of cold plasma is a hydroxyl radical. It seems interesting to study the effect of the products formed under hot plasma radiation on organic substances and compare it with the changes occurring under the action of cold plasma.

Convenient models to study the conditions of proteins are aromatic amino acids contained in these proteins (tryptophan, tyrosine, phenylalanine) since they are identified by absorption bands and characteristic fluorescent lines. The alterations occurring in the structure of the aromatic amino acids immediately manifest themselves in absorption bands and fluorescence [6]. Tyrosine was chosen as a research subject since it and its nitration product (3-nitrotyrosine) are identified by absorption bands, while tyrosine itself — by fluorescence as well [7]. The mechanism of 3-nitrotyrosine formation was studied in the work [8], and the review on tyrosine nitration as part of proteins was reported in the study [9].

**The aim of the investigation** was to study sporicidal activity of pulsed radiation of hot plasma of spark electric discharge by the analysis findings of active products formed in an aqueous L-tyrosine solution under the effect of the discharge. These active products determine chemical reactions.

The study objectives were to compare the changes in a tyrosine solution under cold plasma of a corona electrical discharge and pulsed radiation of hot plasma of spark discharge, and to assess the role of nitration in biocidal and sporicidal effects of hot plasma radiation.

## Materials and Methods

### General conditions

The experiment with L-tyrosine was carried out using two devices generating reactive species: a source of pulsed radiation of hot plasma of spark discharge Pilimin IR-10 (Skobeltsyn Institute of Nuclear Physics, Lomonosov Moscow State University, Russia), hereinafter referred to as IR-10, and a corona electrical discharge reactor. Using them, we treated an L-tyrosine solution in distilled water, its concentration being 160 mg/L (8.8.10^–4^ mol/L), pH 5.65. The absorption bands of the solutions were measured by SF-102 spectrophotometer (Akvilon, Russia); the fluorescence was measured by FLUORAT-02-PANORAMA device (Lumex, Russia); pH — by Expert-001 device (Econix, Russia). For all the experiments, we used analytical grade reagents and distilled water, pH 6.5. The samples were of various volumes; therefore, the dose values were normalized for an actual sample volume (10 or 4 ml of solution).

**Figure 1.**
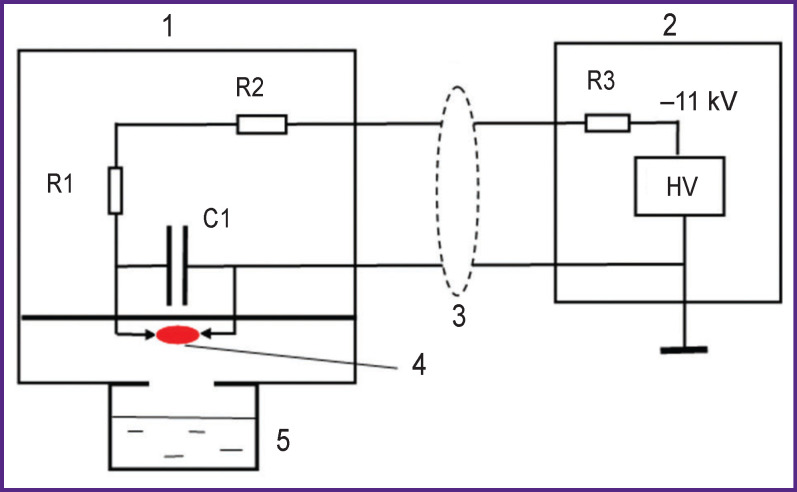
The diagram of pulsed radiation source of hot plasma of spark discharge, Pilimin IR-10: *1* — radiation source module; *2* — electric power supply module; *3* — HF cable, ρ=75 Ω; *4* — discharge area; *5* — Petri dish with treatable sample; R1, R2, R3 are resistors; С1 is energy discharge capacitor; HV is high voltage source

***The source of pulsed radiation of hot plasma, Pilimin IR-10*** was mounted in the form of two modules: a discharge module *1* and an electric power supply module *2* (Figure 1).

In the discharge module *1* made of polytetrafluorethylene, there are stainless steel electrodes, 2-mm thick; between them, a discharge occurs. The distance between the electrodes is 3 mm, which corresponds to the interval sparkover voltage of 6 kV. The electrodes are connected to a capacitor, its capacity being 3300 pF. When there was high voltage (11 kV), a self-sustained spark discharge occurred. A complete current impulse time was 100 μs, the leading edge — 50 ns, the impulse discharge energy — 0.059 J, pulse repetition frequency — 10 Hz, and discharge power — 0.59 J/s. Current impulse time was determined by plasma-discharge time. The current used from the power supply was 0.70±0.02 mA. The discharge yielded ~5% of energy taken from the power supply. Hot plasma radiated as a black body heated to the temperature of ~10^4^ K, the radiation spectrum maximum was at wavelength of 220 nm [2].

The UV radiation intensity of IR-10 generator determined by iodimetric method at a 3-cm distance from the discharge area was (1.26±0.20)**·**10^−10^ mol(cm^2^s)^–1^ [2, 3]. The conditions were chosen so that spark discharge plasma was slightly ionized, the ionization degree being less than 0.1%. Electron density was ~10^11^ cm^−3^, and electron energy did not exceed 1 eV [3, 4].

A part of the module where a discharge occurs was railed off from the other part by an impermeable fluoroplastic membrane. High voltage was imposed to the discharge module via radio frequency cable, its shock wave drag being ρ=75 Ω. A grounded cable cord was directly connected to one capacitor plate, С1. The central cable cord was connected to another capacitor plate through two resistors, R1 and R2 (330 kΩ, 2 W) mounted on the discharge module. Resistors R1 and R2 meant for extinction of reflected waves occurring at the moment of a discharge.

In the electric power supply module *2*, there was a high-voltage rectifier, 11 kV. The zincode of the rectifier was connected through a ballast resistor R3=8 MΩ with a central cable cord. Voltage of 11 kV was imposed to charge а C1 capacitor through the R1, R2, and R3 resistors. A charging circuit time invariable was determined by the value R3×C1  s since R1 and R2<<R3. A discharge cavity through the opening, 2 cm in diameter, was connected to a glass Petri dish, 40 mm in diameter, where the sample of the treated fluid was, its volume being 10 or 4 ml. The distance from the discharge area to the liquid surface was 30 mm. The energy released in a spark discharge within 20 min was 700±40 J per 10 ml of a treated solution.

**Figure 2.**
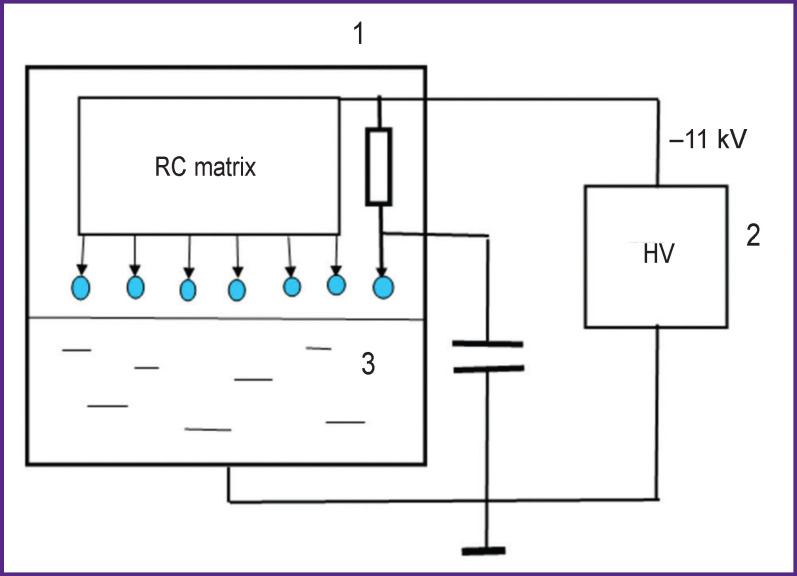
The scheme of sample treatment by cold plasma of corona electric discharge: *1* — body of corona electric discharge reactor; *2* — power supply board; *3* — treatable liquid; HV is high voltage source; RC matrix is matrix of resistors and capacitors

***A corona electrical discharge reactor*** (Figure 2) includes a glass container *1*, its volume being 0.5 L, and it was filled with 50 ml of a treatable fluid sample *3*. The fluid sample was grounded through a hole in the bottom. There were 7 discharge electrodes placed at 6-mm distance from the fluid surface. The electrodes were fastened 25 mm away from each other. Through a set of resistors, total resistance of 20 MΩ (6 resistors 3.3 MΩ each, 2 W), each electrode was imposed high voltage of negative polarity (–11 kV) from the power supply *2*. The discharge current of each electrode was 70 μA, the voltage at each electrode in relation to the fluid considering voltage drop in resistors was 9.6 kV. Each electrode was connected to the earth through a 30-pF capacitor.

The energy released in a corona discharge was 1200±60 and 2400±120 J per 10 ml of a treated solution within 20 and 40 min, respectively.

### UV source

Continuous ultraviolet radiation, 253.7-nm wavelength, was generated by DKB-9 low-pressure mercury lamp, its body made of cathedral glass; the lamp power was 9 W. Power flow density at a distance of 3 cm from the lamp was 2.6**·**10^–2^ J(cm^2^s)^–1^. The energy per fluid sample, 10 ml in volume, was 19.6 J(min)^–1^.

### Tyrosine fluorescence measuring

Tyrosine excitation and fluorescent wavelength are known; however, they were specified in a separate experiment to consider the characteristics of the equipment applied. To determine excitation wavelength, we measured the fluorescence yield at 303-nm wavelength in the range of excitation wavelength 200–300 nm. The maximum fluorescent yield was at the excitation wavelength of 275±1 nm. To determine fluorescent wavelength, we measured fluorescent yield at excitation of 275 nm at a range of wavelength recording 290–320 nm. The maximum fluorescent yield was received at 303±1-nm wavelength recording.

Fluorophores are known to absorb their own radiation. Self-absorption grows with the solution concentration increasing. The study of fluorescence yield dependence on a tyrosine solution concentration within the range of 1 to 20 mg/L showed the fluorescence intensity to linearly grow when the concentration increases up to 12 mg/L, then it levels off and starts decreasing. Therefore, to study tyrosine fluorescence in the present research, we used a tyrosine solution concentration equal to 10 mg/L (treated tyrosine solutions at a concentration of 160 mg/L were 16-fold diluted).

### The treatment of biological samples

The experiment with bacteria and spores of micromycetes was carried out using IR-10 hot plasma generator and DKB-9 low-pressure UV lamp. A biocidal effect was assessed on bacterial strains of antibiotic-resistant gram-positive microorganisms *Staphylococcus aureus* 5913 and gram-negative microorganisms *Escherichia coli* 775-3. The bacterial strains were given by the Museum of the Epidemiology, Microbiology and Immunology Department of Privolzhsky Research Medical University (Nizhny Novgorod, Russia). For analysis, we preliminarily prepared a diurnal culture of microorganisms. Then bacterial cells were resuspended in Hanks’ solution up to the concentration (10–15)**·**10^6^ cells in 1 ml. 4-ml suspension of microorganisms was treated by pulsed radiation of hot plasma of IR-10 generator within a period of time (up to 70 s), and by UV radiation of DKB-9 low-pressure mercury lamp — up to 10 s. A treated suspension was plated on an agar layer and incubated in a temperature-regulated chamber at 37°С within 24 h. A biocidal effect was determined by a CFU (colony-forming units) number.

A sporicidal effect of radiation was assessed on micromycete cultures: *Alternaria alternata* ICM F-1120, *Aspergillus niger* ICM F-1119, *Chaetomium globosum* ICM F-109, *Penicillium chrysogenum* ICM F-245 received from All-Russian Collection of Microorganisms. A spore suspension in water was prepared, its concentration being (2–4)**·**10^6^ cells in 1 ml. The 4-ml volume suspension samples were exposed to radiation using IR-10 generator and DKB-9 lamp within a period of time up to 10 min. The treated suspension was plated with Czapek–Dox medium. The samples were incubated in a temperature-regulated chamber at 27°С within three days; the sporicidal effect was measured by a CFU number.

Sporicidal and biocidal effects of pulsed radiation of hot plasma, and low-pressure mercury lamp products were measured over the sample surface according to a radiation dose. D10 dose represented the 10-fold decrease in CFU number after the treatment (CFU_0_/ CFU_t_=10), where CFU_0_ was the number of CFU in the initial sample; CFU_t_ was their number within a period of time (t) after the treatment.

### Statistical analysis

The experimental data were statistically processed using Microsoft Excel software package. The data are represented as М±m, where М is a mean value, and m is a standard deviation.

## Results and Discussion

### Results of tyrosine treatment by hot and cold plasma radiation

**Figure 3.**
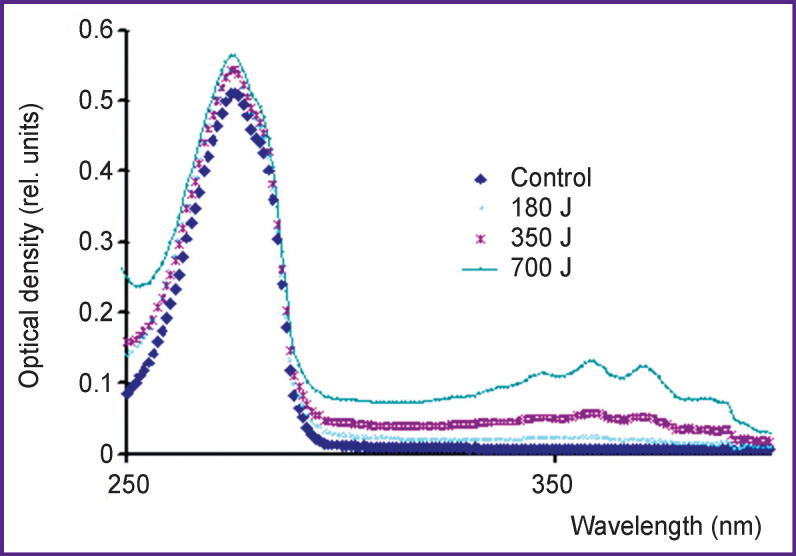
Tyrosine solution absorption bands with concentration of 160 mg/L immediately after treatment by hot plasma radiation of IR-10 generator Radiation doses — 180, 350, and 700 J (per 10-ml solution)

An initial solution of L-tyrosine is neutral, pH 5.7. After the treatment by hot plasma pulsed radiation of IR-10 spark discharge generator, the solution pH decreases: at a radiation dose of 180 J — up to 3.8; at a dose of 700 J— up to 3. Figure 3 shows the tyrosine solution absorption band immediately after the treatment. The characteristics of the main absorption peak related to a phenolic ring, at λ=274 nm, appear not to change much. Within the range of wavelengths 335–385 nm, there are the lines of nitrous acid formed under radiation.

**Figure 4.**
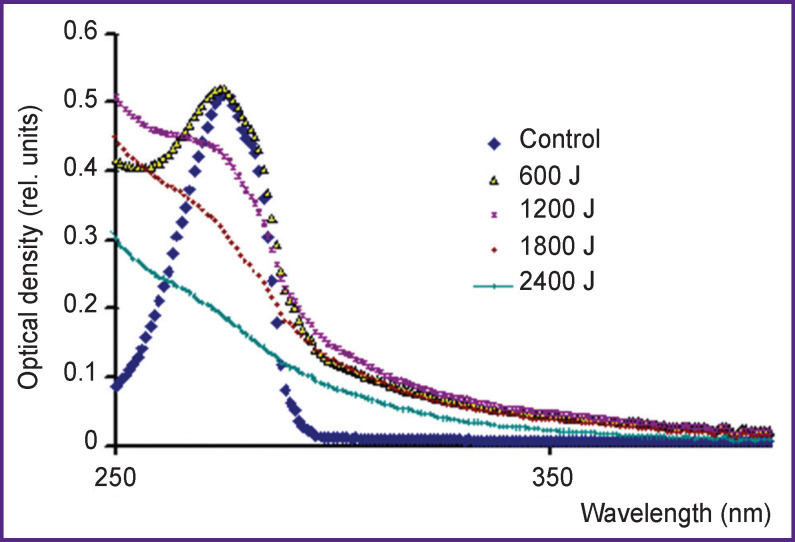
Tyrosine solution absorption bands with concentration of 160 mg/L immediately after treatment by cold plasma of corona electric discharge Radiation doses — 600, 1200, 1800, and 2400 J (per 10-ml solution)

Figure 4 shows the tyrosine solution absorption bands immediately after treatment by cold plasma of corona discharge at radiation doses from 600 to 2400 J. A pH value after treatment unalters. The main absorption peak at λ=274 nm appears to decrease further, and at a radiation dose of 2400 J it completely disappears.

**Figure 5.**
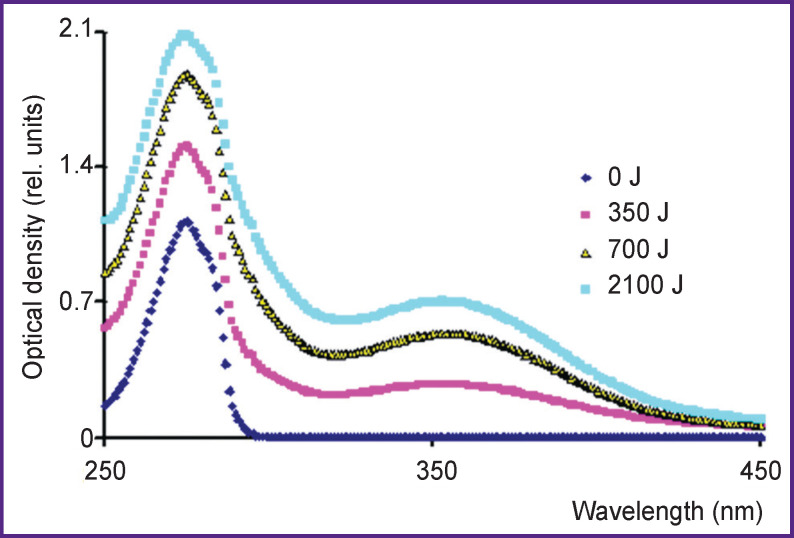
Tyrosine solution absorption bands with concentration of 160 mg/L 24 h after radiation treatment of IR-10 generator Radiation doses — 0, 350, 700, and 2100 J (per 10-ml solution)

Next day, the absorption band of the solution treated by cold plasma of spark discharge does not change. Nitrous acid lines disappear in the spectrum of the solution treated by hot plasma pulsed radiation of IR-10 generator, and a 353-nm peak appears (Figure 5); its optical density grows with a radiation dose increasing. It can be assumed that the peak is related to 3-nitrotyrosine formation. The first day after treatment, crystalline NaOH was added to the acidic solution immediately prior to the spectrum measurement to achieve pH 12 to identify a product.

**Figure 6.**
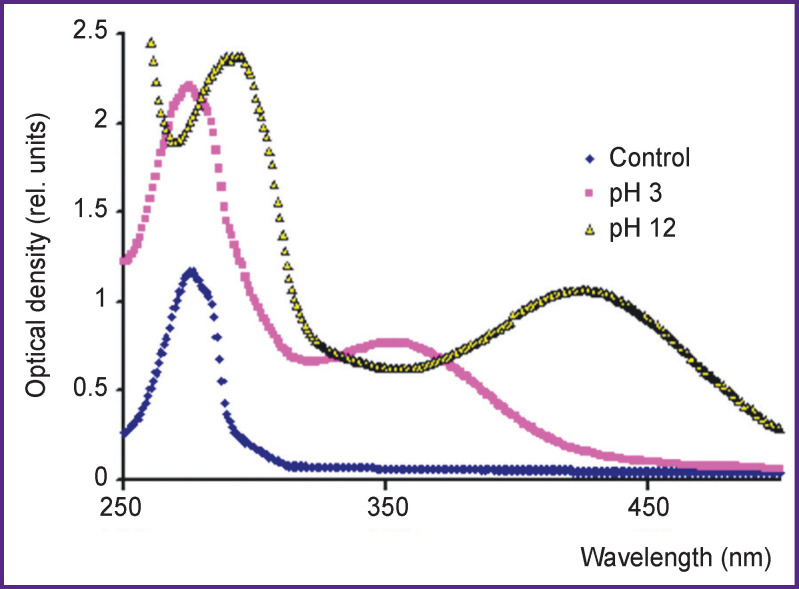
Tyrosine solution absorption bands with concentration of 160 mg/L: initial, 24 h after treatment by hot plasma radiation of IR-10 generator (pH 3), and after adding an alkali (pH 12)

The spectra of the solutions with pH 3 and 12 changed the next day after the treatment using IR-10 generator (Figure 6). The 353-nm peak appears to shift to the right up to λ=425 nm. According to the data of the study [6], 3-nitrotyrosine peak in acidic and alkaline media within the range of possible measurement errors is approximately at the same wavelengths (353 and 425 nm). Thus, a nitration process under hot plasma spark radiation can be considered proved since at least one product of the reaction was found. The identification of other products was beyond the research objectives.

**Figure 7.**
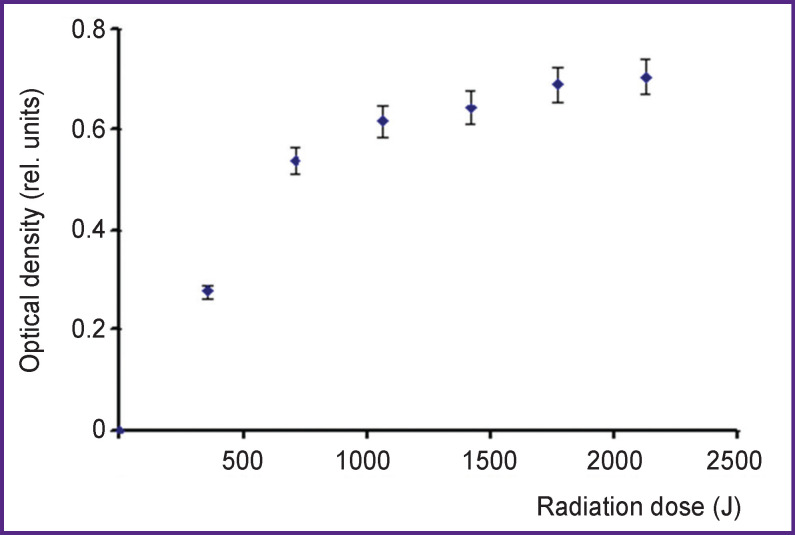
Dependence of optical density on a radiation dose for 3-nitrotyrosine lines (λ=353 nm) 24 h after treatment by hot plasma radiation of IR-10 generator

A nitration product can be found during the first days after treatment and the following days. The addition of alkali shifts a 3-nitrotyrosine peak position. However, if an alkali is added immediately after the treatment, a 3-nitrotyrosine peak appears neither the first, nor the following days. The optical density of 353-nm peak the first day after the treatment by IR-10 generator increases with a radiation dose, and within the range of errors it levels off if a dose is over 1800 J/10 ml (Figure 7).

**Figure 8.**
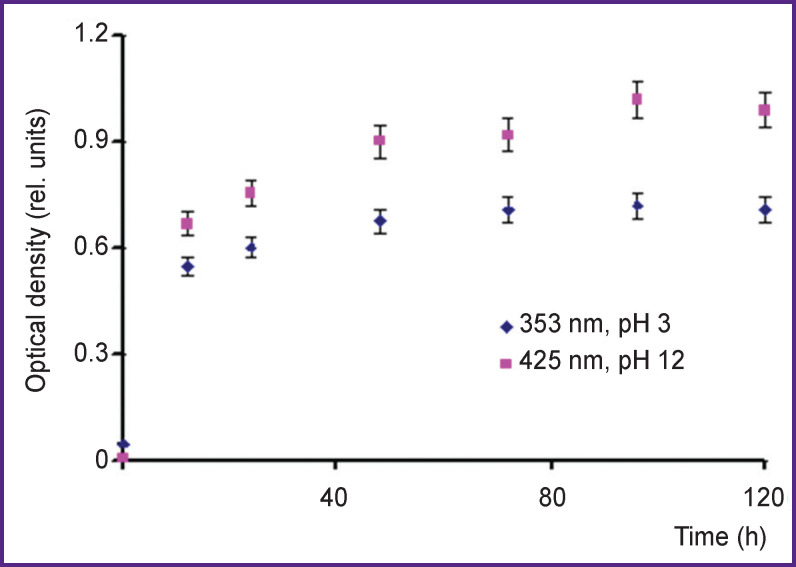
Dependence of optical density for 3-nitrotyrosine lines in an acidic medium (pH 3, λ=353 nm) and in an alkaline medium (pH 12, λ=425 nm) on time after L-tyrosine solution was treated by hot plasma radiation of an IR-10 generator

Nitration with 3-nitrotyrosine formation can occur under the effect of pulsed radiation of hot plasma in the reaction with nitrous acid formed directly in the treatment process, and in the reaction with peroxynitric acids formed at complex …ONOOH/ONOO^–^… breakdown after the treatment. To assess the role of different nitration channels, a subsidiary experiment was carried out. We studied the dependence of optical density of 3-nitrotyrosine peaks in acidic and alkaline media on a time period (up to 120 h) after treatment. An acidic medium was created in a sample directly during the treatment process and preserved during the exposure within the specified time. An alkaline medium was produced by adding NaOH crystals to the treated solution after exposure within a specified period of time, and immediately prior to the measurement of absorption bands. Figure 8 represents the findings for the solutions treated by plasma radiation of IR-10 generator within 20 min at a dose of 700 J/10 ml.

Sodium nitrite NaNO_2_ and sulfuric acid H_2_SO_4_ were added to the solution to study tyrosine nitration using nitrous acid. Nitrous acid resulted from the reaction:


(1) $$2{\text{NaNO}}_{2}+{\text{H}}_{\text{2}}{\text{SO}}_{4}\to {\text{Na}}_{\text{2}}{\text{SO}}_{4}+2{\text{HNO}}_{2^{•}}$$

**Figure 9.**
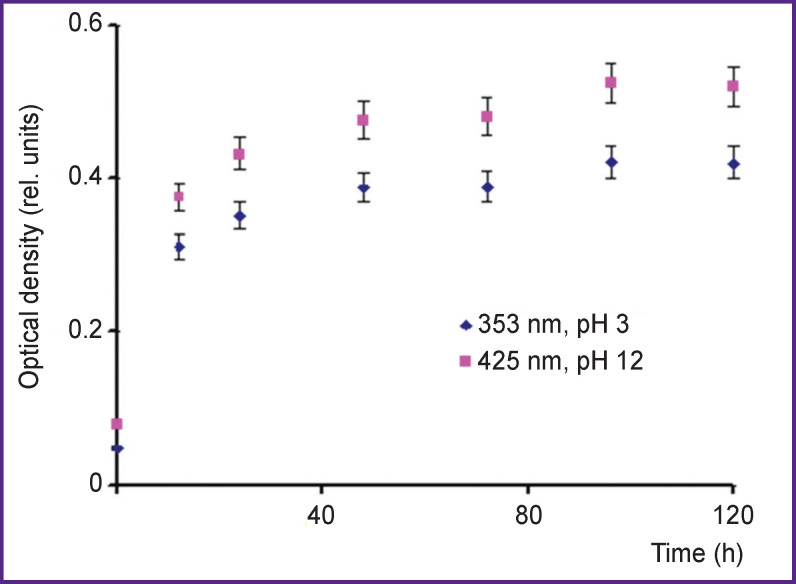
Dependence of optical density for 3-nitrotyrosine lines in an acidic medium (pH 3, λ=353 nm) and in an alkaline medium (pH 12, λ=425 nm) on time after NaNO_2_ and H_2_SO_4_ were added to L-tyrosine solution

Reagents were selected in such a way that the nitrous acid concentration in the reaction (1) was equal to its yield during the radiation by the IR-10 generator within 20 min (a radiation dose of 700 J). Figure 9 shows the dependence of optical density of 353- and 425-nm peaks (in acidic and alkaline media) on time (up to 120 h) after the administration of sodium nitrite and sulfuric acid.

Figures 8 and 9 demonstrate a nitration reaction to proceed slowly, the optical density of 3-nitrotyrosine peak increases with time after treatment, and within the experimental error it achieves the plateau in ~100 h. The comparison of Figures 8 and 9 shows the optical density of 3-nitrotyrosine peak in a solution treated by hot plasma pulsed radiation when nitrous and peroxynitric acids contribute to nitration about twice as much as in the solution with NaNO_2_+H_2_SO_4_ when only nitrous acid contributes to nitration. It suggests that the contribution of nitrous and peroxynitric acids to nitration is nearly the same.

**Figure 10.**
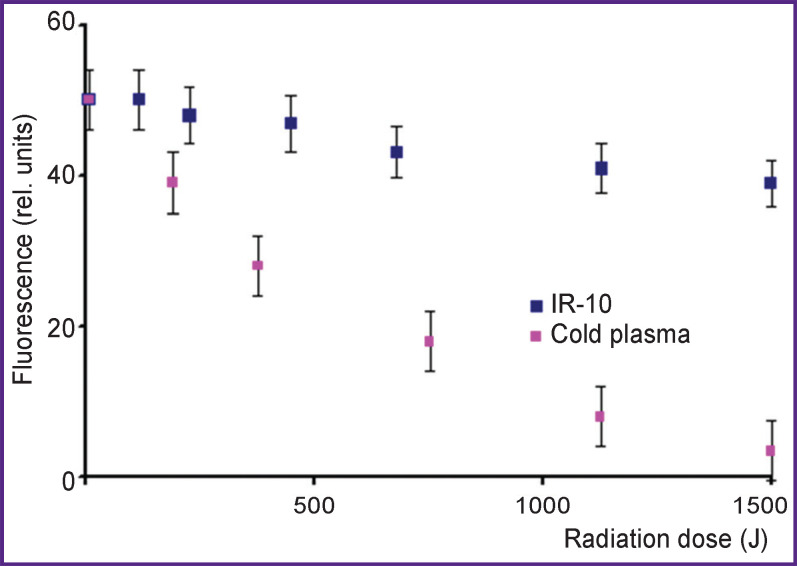
Dependence of tyrosine solution fluorescence on radiation dose J/10 ml 24 h after the treatment by hot plasma radiation of an IR-10 generator and cold plasma of corona electric discharge immediately after treatment

The analysis of the dependence of tyrosine fluorescence on a radiation dose of IR-10 generator 24 h after treatment by cold plasma of corona discharge immediately after the treatment (Figure 10) showed that at a dose of 1000–1500 J/10 ml under corona discharge plasma, tyrosine nearly completely destroys. However, under IR-10 generator radiation at the same dose, the fluorescence decreases by 5–10% and it cannot affect an absorption spectrum anyway. Thus, the data on fluorescence correspond to those on the absorption spectra (see Figures 3 and 4).

### Tyrosine oxidation mechanism

Both oxidizers and deoxidizers are formed under hot plasma radiation of spark discharge in water [4]. Under the corona electric discharge effect, hydroxyl radicals, hydrogen dioxide, and ozone form in water vapors [10]. Radicals HO^•^ and HO_2_^•^ can initiate the oxidation of organic substances. In respect to tyrosine (Tyr), an initiation step can be expressed as follows:

(2) $$\text{TytH}+{\text{OH}}^{•}\to {\text{Tyr}}^{•}+{\text{H}}_{\text{2}}\text{O},k_{1}=10^{9}-10^{10}{\text{mol}}^{-1}{\text{s}}^{-1};$$

(3) $$\text{TytH}+{\text{HO}}_{2}^{•}\to {\text{Tyr}}^{•}+{\text{H}}_{\text{2}}{\text{O}}_{2},k_{2}=10^{3}-10^{5}{\text{mol}}^{-1}{\text{s}}^{-1}.$$

Hereinafter, we use the data on reaction rate constants *k* from the reference book [11]. In the reactions when a hydrogen atom detaches from a TyrH molecule (formula (3)), energy *Е* releases, which is equal to the binding energy of a resultant molecule:

(4) $${\text{OH}}^{•}+{\text{H}}^{•}\to {\text{H}}_{2}\text{O},E=115\text{kcal/mol;}$$

(5) $${\text{HO}}_{2}^{•}+{\text{H}}^{•}\to {\text{H}}_{2}\text{O},E=88\text{kcal/mol.}$$

Hereinafter, we use the data on binding energies from the reference book [12]. The energy released in the reactions (4) and (5) can be used for a hydrogen atom to detach from a molecule. The energy of hydrogen atom detachment in an aromatic ring is 89 kcal/mol [13]. It is evident that aromatic substances can be oxidized by hydroxyl radicals formed in cold plasma of corona discharge and cannot be oxidized by HO_2_^•^ radicals formed in water under pulsed radiation of hot plasma. Figures 3–5 prove these conclusions.

Thus, tyrosine oxidation by primary reactive species formed under plasma radiation appears to be energetically impossible. The secondary decay products of the complex are strong oxidizers, and in peroxynitrite isomerization hydroxyl, radicals form [14]. However, the concentration of the secondary particles is far less than that of the primary ones; and according to the experiment, the role of these secondary particles is small to negligible. Therefore, tyrosine oxidation by hot plasma radiation resulting in aromatic ring destruction does not occur. Thus, the main reactive species determining reactive capacity of radiation are NO_2_^•^ radicals formed at the breakdown of nitrous and peroxynitric acids, which activate a nitration process. Tyrosine nitration was observed in the present experiment.

### Nitration mechanism

The main reactive species initiating nitration in the present experiment are nitrous acid formed directly under radiation and peroxynitric acid formed when the complex …ONOOH/ONOO^–^… decomposes, which forms under radiation and breaking down within a period of time up to 15 days [2, 3]. A nitration process can be representing as initial and final conditions as follows:

(6) $$\text{TyrH}+{\text{NO}}_{2}^{•}\to {\text{TyrNO}}_{2}+{\text{H}}^{•}.$$

The binding energy of hydrogen atom in an aromatic ring of tyrosine is ~89 kcal/mol, the binding energy of NO_2_ group rejoined at the place of hydrogen atom is ~19 kcal/mol. Therefore, a nitration reaction is exothermic, the released energy is ~70 kcal/mol [12]. Energetically, 3-nitrotyrosine formation is most likely [6-9].

In literature, two possible reaction mechanisms are under discussion: radical, and through the formation of nitronium ion NO_2_^+^ A radical mechanism is possible if a tyrosine radical Tyr^•^ forms under radiation. Then a radical NO_2_^•^ can attach to it:

(7) $$\text{TyrH}+\text{hv}\to {\text{Tyr}}^{•}+{\text{H}}^{•};$$

(8) $${\text{Tyr}}^{•}{}^{•}+{\text{NO}}_{2}^{•}\to {\text{TyrNO}}_{2^{•}}.$$

Another variant of a radical mechanism is possible if peroxynitric acid decomposes into HO^•^ and NO_2_^•^ radicals. Tyrosine radical forms when tyrosine molecule interacts with a hydroxyl radical followed by NO_2_^•^ radical attaching to the place:

(9) $$\text{TyrH}+{\text{OH}}^{•}\to {\text{Tyr}}^{•}+{\text{H}}_{\text{2}}\text{O};$$

(10) $${\text{Tyr}}^{•}+{\text{NO}}_{2}^{•}\to {\text{TyrNO}}_{2}.$$

As a matter of principle, such mechanism is possible since both radicals form at one place, when one molecule of peroxynitric acid breaks down [14].

An alternative communication channel with the formation of nitronium ion NO_2_^+^ a nitronium ion forms in an acidic medium in case of acidic remnants of nitric or nitrous acids presence. The process can be represented as follows:

(11) $${\text{HNO}}_{2}+{\text{HNO}}_{3}\to {\text{NO}}_{2}^{+}.$$

Further, a transient condition forms:

(12) $$\text{Tyr}+{\text{NO}}_{2}^{+}\to \text{Tyr}A.$$

When a transient condition breaks down, energy *А* releases, and a nitration end product forms:

(13) TyrA→3-nitrotirosine.

The formation of a transient condition is a slow stage. According to experimental data, it can last 2–3 days.

To identify a nitration product, an alkali was added to the solution exposed at last a day after being treated by spark discharge of hot plasma. If an alkali was added immediately after the treatment, there were no changes observed in the absorption band of the solution 1–2 days later. No 3-nitrotyrosine peak appeared. So, two conclusions can be made. A nitronium ion does not form in an alkaline medium, therefore, a nitration mechanism through a nitronium ion is quite possible. In an alkaline medium, peroxynitrite lifetime is larger than that of peroxynitric acid in an acidic medium. Therefore, the end products of its breakdown, HO^•^ and NO_2_^•^ radicals, should form. However, a nitration product does not appear. Thus, a radical nitration mechanism in the study process is impossible.

### Sporicidal and biocidal effects of plasma radiation of IR-10 generator and DKB-9 UV radiation lamp

The findings of the experiment with bacterial strains and micromycete cultures are presented in the Table.

**Table:** Sporicidal and biocidal effects of the products of pulsed radiation of hot plasma and a low-pressure mercury lamp (M±m)

Cell type	IR-10 (hot plasma)		DKB-9 UV lamp		CFU_0_/CFU_t_
	Dose (D10) (J)	Treatment time	Dose (J)	Treatment time	
*Staphylococcus aureus*	29±3	50 s	1.6±0.2	5 s	10
*Escherichia coli*	41±5	70 s	0.7±0.1	2 s	10
*Aspergillus niger*	280±25	8 min	200	10 min	0.97±0.10
*Alternaria alternata*	177±16	5 min	200	10 min	0.88±0.10
*Chaetomium globosum*	35±4	1 min	200	10 min	0.90±0.10
*Penicillium chrysogenum*	210±23	6 min	200	10 min	0.65±0.15

Note: D10 dose is a 10-fold decrease in CFU number after treatment.

The mechanisms of biocidal and sporicidal effects differ radically. Bacterial cells die after DNA molecules uptake UV radiation energy with wavelength in the range of 250–260 nm. A UV lamp generates radiation with 253.7-nm wavelength; thereby, nearly all radiation energy is accounted for the absorption maximum by a DNA molecule. At the same time, an average flow of UV radiation photons generated by a spark discharge of IR-10 generator is far less compared to that of a UV lamp. And the maximum of infrared radiation of spark discharge is accounted for wavelength of 220 nm, i.e. missing DNA absorption band maximum. Therefore, the energy needed to 10-fold decrease CFU of bacterial cells *Staphylococcus aureus* and *Escherichia coli* under hot plasma radiation is much higher (29±3 and 41±5 J, respectively) than under UV lamp radiation (1.6±0.2 and 0.7±0.1 J, respectively).

However, the instantaneous density of a generator IR-10 at the moment of discharge spark is vastly greater than the instantaneous density of mercury lamp radiation. High instantaneous density of radiation results in the formation of …ONOOH/ONOO^–^… complex in an aqueous solution, it decomposes within a period of time up to 14 days into peroxynitrite and peroxynitric acid [2, 3]. Since under hot plasma radiation an aqueous solution obtains acid reaction, then the main cleavage product of the complex is peroxynitric acid. The cleavage of peroxynitric acid results in the formation of products initiating nitration.

Micromycete spores are covered with a radiopaque multilayer shell. Therefore, UV radiation of a mercury lamp has a mild exposure on spores. The Table demonstrates the 200-J dose of mercury lamp radiation leads to 10% decrease of CFU, while a 200-J dose of pulsed radiation of hot plasma results in 10-fold decrease in CFU. According to the experiment carried out, the oxidation of compound organic substances by products forming under hot plasma radiation can appear unlikely or impossible. A long-living …ONOOH/ONOO^–^… complex diffuses through a protective membrane [14, 15] and when decomposing, it initiates nitration. Thus, nitration can serve the main mechanism causing and determining a disinfecting effect of pulsed radiation of hot plasma.

## Conclusion

The study showed that the viability of spores under pulsed radiation of hot plasma decreases. The radiation of DKB-9 UV lamp under studied conditions slightly penetrates a spore protective membrane. A sporicidal effect of pulsed radiation of hot plasma is due to the cleavage of a long-living …ONOOH/ONOO^–^… complex with the formation of nitrogen oxide and nitronium ion in an acidic medium.

Nitration plays a decisive role in a sporicidal effect of hot plasma spark radiation. The principle of sporicidal effect of gas discharge plasma based on nitration can be used to develop medical disinfecting devices.
